# Lessons of the Israeli-Palestinian Conflict for Public Health: The Case of the COVID-19 Vaccination Gap

**DOI:** 10.3390/ijerph182111292

**Published:** 2021-10-27

**Authors:** Yara Dahdal, Nadav Davidovitch, Michael Gilmont, Javier Lezaun, Maya Negev, Deborah Sandler, Mohammed Shaheen

**Affiliations:** 1 Nature Palestine Society, Ramallah 9993900, Palestine; yaradahdal@gmail.com; 2 School of Public Health, Faculty of Health Sciences, Ben Gurion University of the Negev, Beer Sheva 84105, Israel; nadavd@bgu.ac.il; 3 Institute for Science, Innovation and Society, Oxford Martin School, University of Oxford, Oxford OX2 6PN, UK; michael.gilmont@insis.ox.ac.uk; 4 Institute for Science, Innovation and Society, School of Anthropology and Museum Ethnography, University of Oxford, Oxford OX2 6PN, UK; javier.lezaun@insis.ox.ac.uk; 5 School of Public Health, University of Haifa, Haifa 31905, Israel; 6 Arava Institute for Environmental Studies, Kibbutz Ketura, Hevel Eilot 88840, Israel; deborah.sandler1@gmail.com; 7 Oxford Martin School, University of Oxford, Oxford OX1 3BD, UK; 8 Damour for Community Development, Ramallah 6063139, Palestine; mshaheen2009@gmail.com

**Keywords:** conflict, COVID-19, climate change, one epidemiological unit, MENA, Israel, Palestine, cooperation, non-cooperation, health systems

## Abstract

In early 2020, the COVID-19 pandemic revealed a faceless, non-adversarial threat that endangered Israelis and Palestinians with the same ferocity. However, the capacities of the health systems to address it were not equal, with Israel more equipped for the outbreak with infrastructure, resources, manpower and later, vaccines. The pandemic demonstrated the life-saving benefits of cooperation and the self-defeating harms brought by non-cooperation. These trends are explored here by an international team of public health and environmental scholars, including those from different sides of the Israeli–Palestinian conflict. This article explores the importance of recognizing the Israeli and Palestinian jurisdictions as a single epidemiological unit, and illustrates how doing so is a pragmatic positioning that can serve self-interest. We demonstrate how despite political shocks precipitating non-cooperation, there has been a recurrent tendency towards limited cooperation. The paper concludes with lessons over the need for reframing public health as a potential bridge, the need for structural changes creating sustainable platforms for accelerated transboundary cooperation to enable the steady management of current and future public and environmental health crises regardless of dynamic political crises, and the importance of civil society and international organizations in forging collaboration in advance of governmental engagement.

## 1. Epidemiological Co-Dependencies and Political Divides

In June 2021, when the COVID-19 vaccination rate in Israel was above 60%, and in Palestine below 10%, the two governments signed an agreement to transfer vaccines from Israel to the Palestinian Authority. This transfer was defined as a loan, which would be repaid with a shipment of vaccines which the Palestinian Authority was due to receive in the future. When the vaccines arrived from Israel, they were found to have a short expire date. They were returned by the Palestinian Authority, and the original agreement was cancelled [1]. This incident came to exemplify the lack of trust between the two entities, even when fundamental values for both parties, such as human rights and public health, are at stake. In order to effectively protect both populations, as is the case with much public health management [2], the pandemic required a degree of trust between Israeli and Palestinian officials, as well as concerted and consistent cooperation amidst tense and shifting political conditions. Since the pandemic started in March 2020, a limited degree of cooperation has taken place between the governments, public health authorities and non-governmental organizations (NGOs). This has included the exchange of epidemiological information and disease outbreak data, lessons on medical management, transfer of vaccines, and the vaccination of cross-border populations. However, these examples of co-ordination have unfolded against a background of periods of diplomatic disengagement and also overt war. This non-cooperation has not only stunted progress in the control of COVID-19, but also increased the magnitude of the task at hand, by creating information gaps, facilitating the spread of disease, delaying the roll-out of the vaccines, and diverting political attention from the task of pandemic management. COVID-19 has therefore demonstrated the life-saving benefits of cooperation and the self-defeating harms brought by non-cooperation. These trends are explored here by an international team of public health and environmental scholars, including those from different sides of the Israeli–Palestinian conflict.

The political tension is manifested, among other things, by Israeli control over Palestinian borders, movement and land. These are crucial aspects for any pandemic control, on top of imbalance of power, inequity of resources and a stressed, fragmented Palestinian healthcare system. These characteristics make cooperation more difficult and unpredictable. The initial cross-border response to the crisis offered unexpected examples of ad hoc assistance between the Israeli Government and the Palestinian Authority, underpinned by the severity of the threat [3,4]. Starting in March 2020, this included limited exchanges of medical supplies, funding and medical training. As a result of strict national policies across the region, and a degree of cooperation between the parties, the infection rates were kept relatively low (less than 100 new reported cases per country [5]) in both Israel and the Palestinian territories, which contained the impact of the virus. In July 2020, however, the Trump administration plan for the region and the subsequent announcement by the Israeli Government to proceed to the annexation of 30% of the West Bank led the Palestinian Authority to boycott any kind of cross-border cooperation, including urgent activities focused on managing the pandemic, thus, ending a cautiously hopeful period of pragmatic cross-border cooperation.

**Figure 1:**
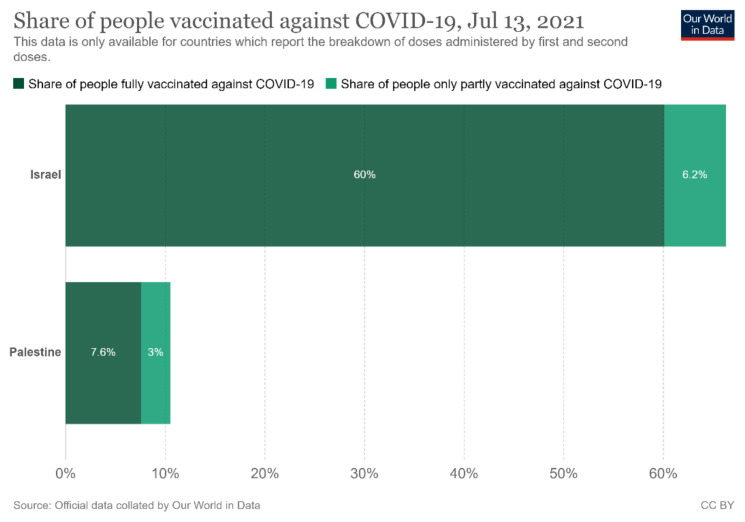
Share of People Vaccinated Against COVID 19, 13 July 2021 for Israel and Palestine [8], used under Creative Commons License CC BY 4.0.

The failures precipitated by this breakdown in cooperation were brought into focus in late 2020 by the stark contrast between the immunization rates of the Israeli and Palestinian populations, still evident at the time of writing (see Figure 1). The vaccination gap brought into sharp relief the challenge of sustaining joint efforts in a region that is unified by shared epidemiological risks and realities but remains chronically divided by long-standing political conflicts [6]. While Israel has offered the world an example of a rapid and effective vaccine roll-out, across the border vaccination rates among Palestinians within the West Bank and Gaza have continued to lag. There have been unheeded calls on both sides to create or allow minimal spaces of collaboration to safeguard the health of all the citizens in what are effectively deeply interconnected [7].

Resumption of cooperation in early 2021 included the transshipment of vaccines into Gaza and the West Bank, and vaccination of Palestinian workers living in the West Bank working in Israel. This period of cooperation was again stalled in May 2021. By the time of the outbreak of the violent May 2021 conflict, most of the eligible Israeli population was vaccinated, compared to a fraction of the Palestinian population (60% vs. 5% first doses [6]), leaving a significant element of the regional population vulnerable to continued COVID-19 spread. The lessons of COVID-19 cooperation and non-cooperation are important not only for management of the current pandemic, but also for informing management of future health and environment-related risks, compounded by climate change [9], where cooperative management will be required to protect the entire population of the region, regardless of the transient state of politics. This would require providing significant support to Palestinians in building the capacity of their healthcare system, and providing more control over their jurisdictions, movement and resources. Such support would also enhance trust and achieve more equitable and sustainable cooperation between the two sides. Under current arrangements, the gross economic disparity and power between the two sides will result in an ineffective regional immunization. Consequently, the regional population immunity will remain a distant prospect, opening up several opportunities for the virus to continue to spread and mutate [10].

Cooperation between Israel and the Palestinian Authority, their medical and public health services, with particular reference to preventive measures to combat the spread of epidemics, should be based on human rights declarations, public health considerations and international legal duties. Moreover, there is precedent for regional health management and treating the region as a single epidemiological unit. Between the 1967 war and the 1995 Oslo Accords, health services in the West Bank and the Gaza Strip were the responsibility of the Israeli civil administration, as the occupying power. During this period, Israel recognized the importance of routinely vaccinating the Palestinians, both to meet its responsibilities under international law and for its own self-interest. This was the case, most noticeably, for maternal and child vaccination programs. The public health benefits of these policies were evident. The control of poliomyelitis, for example, was only achieved in the 1970s once Israeli ramped up inoculations in the West Bank and Gaza Strip [11]. In-contrast, political unrest in Syria led to the re-emergence of tuberculosis, cutaneous leishmaniasis, polio, cholera, and measles in the country as well as in neighboring Turkey, Lebanon, Jordan and Iraq [12]. Similarly, the political unrest in Afghanistan and Pakistan hindered polio eradication efforts [13]. Likewise, civil war and political unrest in different African countries facilitated the spread of Ebola [14]. 

Even after the Oslo Accords devolved public health responsibilities to the Palestinian Authority, examples of effective coordination continued to show the value of treating Israel and Palestine as one epidemiological unit. In 2009, the Israeli civilian administration assisted in Palestinian immunization efforts to control H1N1 (swine flu) by facilitating the transfer of 25,000 vaccine doses donated by the International Committee of the Red Cross. The COVID-19 crisis has, however, revealed the shortcomings of ad hoc coordination, and the limits that lack of progress on the underlying political conflict places on the prospects for governmental and non-governmental cooperation. Regardless of legal disputes over the Palestinian Authority’s health obligations under the Oslo Accords and Israel’s responsibilities as an occupying power under the Fourth Geneva Convention, it is evident that both Palestinian and Israeli citizens have an enlightened self-interest in effective transboundary control of COVID-19 and indeed any other, present or emerging, infectious disease [15].

Despite the devolution of public health responsibilities to the Palestinian Authority, Israel retains a significant level of control that can determine the ability of Palestinians to protect their own health. According to the Oslo Accords, for example, all medical supplies, including vaccines, must be licensed by the Israeli government before reaching the Palestinians. Lack of Palestinian control over their borders has resulted in multiple delays in the roll-out of vaccines; an early shipment of Russian Sputnik V vaccines was delayed entry to Gaza by Israel in February 2021, as supplies got caught up in the wider political conflict [16]. Palestinian challenges are compounded by a limited cold storage infrastructure, including only one suitable freezer in the West Bank.

Likewise, inadequate information sharing between Israel and the Palestinian Authority has been an unnecessary and damaging factor. Sharing clinical epidemiological data, including on emerging variants of concerns, should be of mutual interest for both Israelis and Palestinians. The benefits of information sharing were evident during the initial phase of the pandemic response, but it became another victim of the deteriorating political situation. Subsequent pressure from the international community, and from within Israel itself, has recently led to a resumption of cooperation on this and other fronts. In March 2021, Israel transferred 5000 vaccines to the Palestinian Authority, and had vaccinated Palestinians detained in Israeli prisons, and 105,000 Palestinians working in Israel [17]. In February 2021, a survey conducted by the Palestinian Ministry of Health, the Palestinian National Institute of Public Health, and the Palestinian Center Bureau of Statistics showed that 40% of the Palestinian population had coronavirus related antibodies [18]. As of March 2021, the Palestinian Authority has started a concerted vaccination drive, including provision of further vaccine doses through COVAX that have been transferred successfully via Israeli ports [19]. By late June, 482,000 Palestinians had had at least one dose of vaccine, of which 65,000 were in Gaza, which significantly lags the West Bank in vaccination rates (Palestinian Ministry of Health data, 28 June 2021). UAE-sponsored vaccine supplies have also entered Gaza via the Egyptian border, demonstrating the importance of wider regional cooperation in managing the pandemic. The one million doses of Israel’s ageing Pfizer stock of vaccines that were included in the June 2021 vaccination swap deal, announced between the Israeli and Palestinian governments, were a promising means to increase the vaccination rates in the West Bank. However, the cancellation of the deal is testament to the mistrust between the two governments [1].

Arab-Israelis, comprising approximately 20% of the population in Israel, have played a role in helping Palestinians and providing a platform for coordination between the two sides. In December 2020, 12 Arab-Israeli doctors from “Physicians for human rights in Israel” visited Gaza for three days, focusing on urgent cases that had been side-lined as COVID-19 patients filled local hospitals [20]. Arab-Israelis make up 17% of doctors, 25% of nurses, and 50% of pharmacists in Israel and therefore have an important role to play in fostering regional cooperation in public health [21].

## 2. Conclusions

The COVID-19 pandemic has brought national health systems and economies to the brink, as governments all around the world struggled to contain the virus and safeguard the wellbeing of their populations. In Israel and Palestine, the virus has also brought into stark focus the impact of long-standing political conflict and policy shortcomings on the ability of both governments to mount effective public health action. As a result, the pandemic has exacerbated chronic forms of precarity in those groups that are most vulnerable in the ongoing conflict. Yet Israel and Palestine are too intertwined and interconnected to exist in separate epidemiological realities. An effective pandemic response required greater integration of regional politics, policies and resources.

The impact of COVID-19 and the vaccination roll out has clearly illustrated the short- and long-term risks posed by the lack of effective cooperation. The authors are not so naïve as to expect that protracted political conflict and chronic policy challenges will suddenly disappear in the face of a common threat, even one as acute and systemic as the COVID-19 pandemic. However, we suggest that the cross-border nature of infectious diseases is a wakeup call for politicians and health policymakers to consider the political determinants of health more prominently. While it is now widely accepted that social determinants have a crucial role in shaping our health, we are much less engaged in a better understanding of what shapes those social determinants. The importance of cross-border considerations, and the politics that shape them, must be taken more seriously when framing policymaking and research, for example involving stakeholders from all sides in coordinating a health emergency response and building sustainable structures to enable it. We expect all parties to draw a series of useful lessons from their current predicament. This would help improve our collective response to the current crisis and create the basis for more effective action that will help mitigate looming health and environmental crises we face in the future. It is telling that despite the political shocks of recent months and the seemingly intractable political obstacles, there has been a progressive, if slow-moving, shift towards cooperation between Israeli and Palestinian institutions, even if driven primarily by enlightened self-interest.

### Recommendations

We propose the following lessons can be drawn:

1. Policymakers and politicians need to respect and protect the basic human right to health of all people in the region, regardless of their nationality or background. They need to acknowledge that regional political and economic imbalances compromise the efforts to protect the health and wellbeing of their own people.
2. Health systems in the region, especially public health systems, need to accelerate current plans to integrate cross-sectoral cooperation within and across countries, in order to build a more resilient capacity to anticipate, mitigate and respond to current and future crises. This includes regionalizing and globalizing health and climate change strategies, strengthening dialogue and cooperation among state and non-state actors at different levels of governance. The pandemic has demonstrated that even wealthy countries are unable to secure the health of their populations without effective and equitable cross-border cooperation.
3. To this end, and despite the protracted conflict, Israelis, Palestinians and their neighbors need to build a sustainable regional coordination program and/or channel of dialogue independent of, and resilient to, fluctuations in political conditions. The program should be focused on research and improved strategic responses into national and regional planning for various aspects of public health and medicine. Its scope should include sharing information, research findings, experiences and competencies, clinical aspects, technology and resources (including vaccines) to better manage current and future public health crises. Such a program should be composed of health care and operational professionals, with support from the WHO, and should draw upon the tangible gains seen from cooperation early in the pandemic.
4. In situations of chronic political conflict, such as that between Palestinians and Israelis, scientists, public health professionals, human rights advocates and other non-state actors need not wait for their governments to initiate cooperation. In fact, civil society on both sides of the border must work together to redouble cross-border efforts in order to offset the obvious political barriers to achieving universal access to health and security.
5. International organizations including United Nations, World Health Organization, Red Cross, and Doctors Without Borders,) have an important role to play in bridging the collaboration gap among scientists and professionals in the two countries. This includes advocacy for evidence-based policies and programs that address the inseparably intertwined public and environmental health of the people in the region. Such international assistance can have a significant effect in ameliorating the effects of an acute and seemingly intractable political conflict.
6. In cases of pandemic breakouts, the Palestinian Authority should have the power to make independent decisions to allow quick access to vaccines and relevant supporting infrastructures. These actions can be facilitated by international organizations.
7. None of the above recommendations will work without acknowledging the lack of trust and its negative implications for containing a pandemic such as COVID-19. International organizations, governments and health policymakers should work to build the cross-border trust that is the foundation of any collaboration, particularly in conflict zones.

## Data Availability

This study did not report any data.
